# Tricuspid valve agenesis: a case report of a rare cause of tricuspid regurgitation in an adult

**DOI:** 10.1093/ehjcr/ytae075

**Published:** 2024-02-09

**Authors:** Victor C Raicea, Oana Mirea, Mihaela C Berceanu, Andrei D Raicea

**Affiliations:** Department of Cardiovascular Surgery, University of Medicine and Pharmacy of Craiova, Petru Rares nr. 2, 200349 Craiova, Romania; Department of Cardiology, University of Medicine and Pharmacy of Craiova, Romania Petru Rares nr. 2, 200349 Craiova, Romania; Department of Cardiovascular Surgery, University of Medicine and Pharmacy of Craiova, Petru Rares nr. 2, 200349 Craiova, Romania; Clinic of Cardiovascular Surgery, Emergency Institute for Cardiovascular Diseases and Transplantation, Str. Gh. Marinescu nr. 50, 540136 Târgu Mureş, Romania

**Keywords:** Tricuspid valve agenesis, Posterior tricuspid leaflet, Congenital tricuspid regurgitation, Case report, Surgical tricuspid valve replacement

## Abstract

**Background:**

Tricuspid valve agenesis is an exceptionally rare congenital cardiac disease characterized by the incomplete formation or complete absence of one or more tricuspid leaflets. It is commonly diagnosed during childhood due to the development of heart failure symptoms.

**Case summary:**

We report the case of a 62-year-old woman admitted for a worsening of heart failure symptoms (New York Heart Association functional Class IV at admission). A standard transthoracic echocardiogram was performed, which showed severe right heart dilatation and severe tricuspid regurgitation. A three-dimensional echocardiogram revealed the absence of the posterior tricuspid leaflet. The patient underwent an uncomplicated surgical bioprosthetic valve replacement with a favourable outcome and partial recovery of right ventricular function. In our patient, valve repair was not favoured due to the additional mild hypoplasia of the anterior leaflet. The 3-month post-operative evolution of the patient was favourable, with significant symptom relief.

**Discussion:**

The diagnosis of tricuspid agenesis during adulthood is uniquely uncommon. A three-dimensional echocardiography can provide accurate pre-procedural insight into the valve anatomy, allowing surgeons to plan for either valve repair when the anatomy is suitable or valve replacement.

**Learning points**

- Tricuspid valve (TV) agenesis is a uniquely rare cause of congenital tricuspid regurgitation in adults.
- A pre-operative three-dimensional echocardiography can provide decisive data regarding the morphology of the TV.
- Despite the long-standing severe tricuspid regurgitation, surgical intervention was uneventful in the case of our patient in this study and was associated with significant clinical improvement.

## Introduction

The organic (primary) aetiology of tricuspid regurgitation (TR) is reported in a fairly small percentage of patients (8–10%).^1,2^ Nonetheless, this percentage may not entirely reflect reality, since the diagnosis of organic TR requires a correct assessment of the anatomy of the tricuspid valve, which is not always possible with the conventional two-dimensional echocardiography.

The most common causes of organic TR are infective endocarditis, interference from defibrillator leads, myxomatous degeneration, rheumatic fever, and congenital abnormalities.^3–5^

Congenital TR is an uncommon finding and is often diagnosed *in utero* or during early childhood. Moreover, while the current guideline for the management of congenital heart disease^6^ provides certain treatment recommendations for severe TR in the context of Ebstein’s anomaly, there is no explicit guidance on the management of TV agenesia.

## Summary figure

**Figure:**
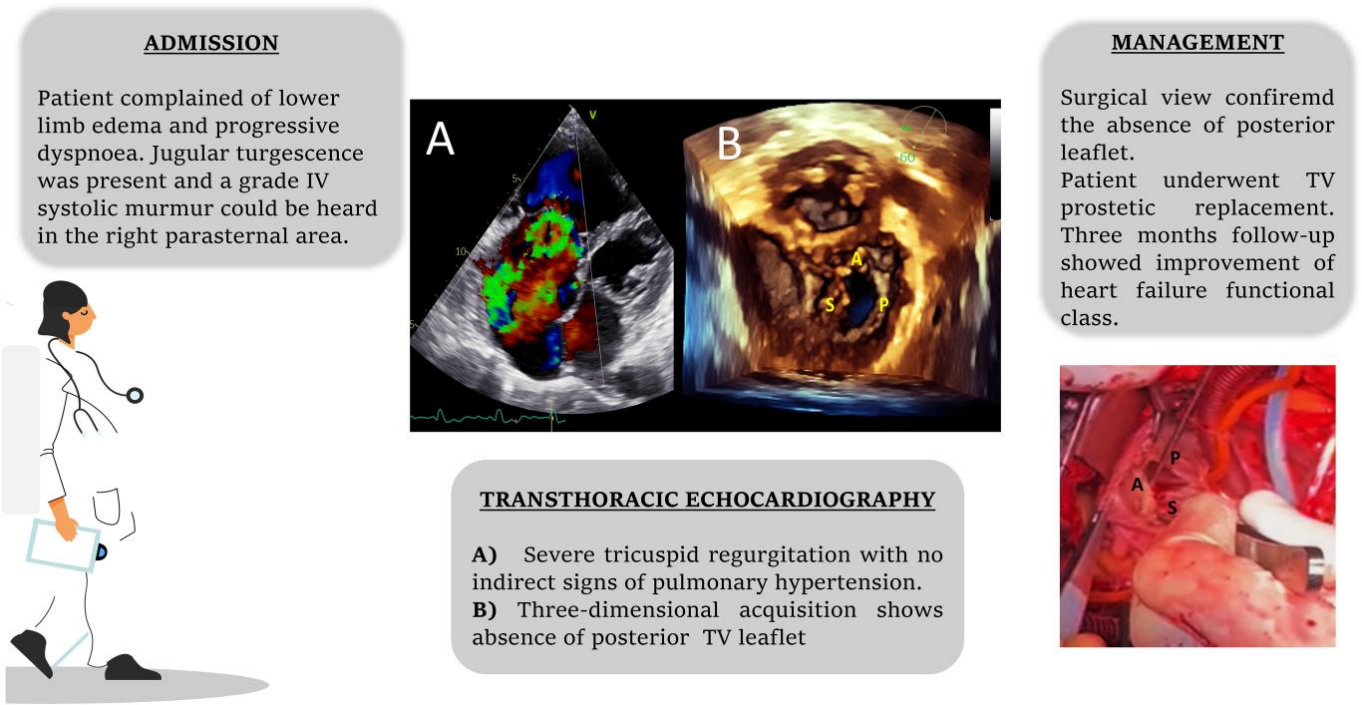


## Case summary

A 62-year-old Caucasian woman with a history of heart failure was admitted to our county hospital in the cardiology department for worsening dyspnoea and lower limb oedema. The symptomatology has progressed slowly over the past few months.

Although her medical history showed records of severe TR with unknown aetiology and permanent atrial fibrillation, the patient did not take regular cardiological examinations.

The home medications included nebivolol (5 mg o.d.), furosemide (40 mg o.d.), spironolactone (25 mg o.d.), and acenocumarol (2 mg o.d.).

The patient was normoponderal (body mass index = 23.1 kg/m^2^) and an active smoker. Upon admission, her vital signs were stable, with blood pressure ranging between 100 and 110/60–70 mmHg, a heart rate of 110–115 b.p.m., and an oxygen saturation (SpO_2_) of 95% on room air. A physical examination revealed the presence of a 4/6 holosystolic murmur heard loudest at the right left sternal border, prominent bilateral jugular turgescence, moderate lower peripheral oedema, and hepatomegaly. Auscultation revealed normal lung sounds.

An electrocardiogram showed atrial fibrillation, 115 b.p.m., incomplete right bundle branch block, and non-specific repolarization abnormalities. A chest X-ray showed normal lung fields and mild cardiomegaly.

A biochemical analysis showed a mild increase in the total bilirubin level (1.28 mg/dL) and serum aminotransferase (40 U/I) and a slightly lower serum K concentration (3.4 mmol/L).

**Figure 1:**
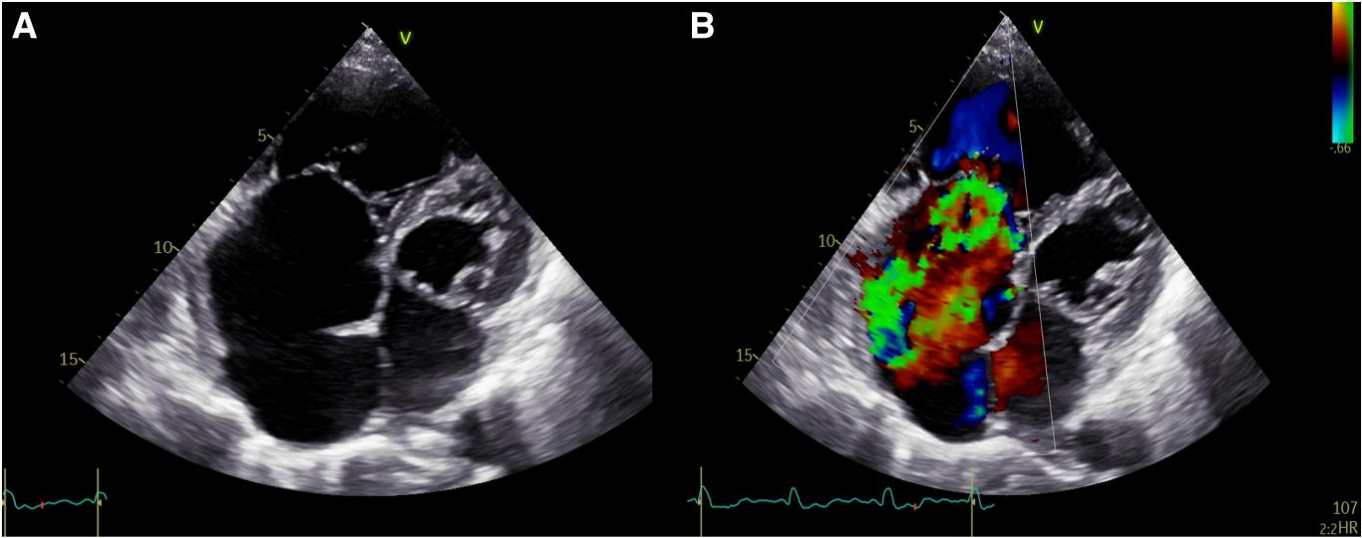
A bidimensional transthoracic echocardiogram shows (*A*) severe right heart dilatation and (*B*) severe tricuspid regurgitation on colour Doppler imaging.

A severe dilatation of the right ventricle at end diastole (56 mm) and of the right atrium at end systole (74 mm, area of 43 cm^2^; *Figure 1A*; Supplementary material online, *Video S1*) and paradoxical ventricular septal motion were observed on a transthoracic echocardiogram. The tricuspid annular plane systolic excursion (TAPSE) was 16 mm, suggesting a mild impairment of right ventricular (RV) function.

Severe TR (vena contracta = 11 mm) secondary to the lack of leaflet co-optation could be seen on colour Doppler imaging (*Figure 1B*; Supplementary material online, *Video S2*). A continuous wave Doppler showed a triangular TR Doppler profile.

The left ventricular ejection fraction was normal (65%), and no abnormal intra-cardiac shunts were observed. The main pulmonary artery diameter was 32 mm. Also, no additional significant valvular disease was observed.

**Figure 2:**
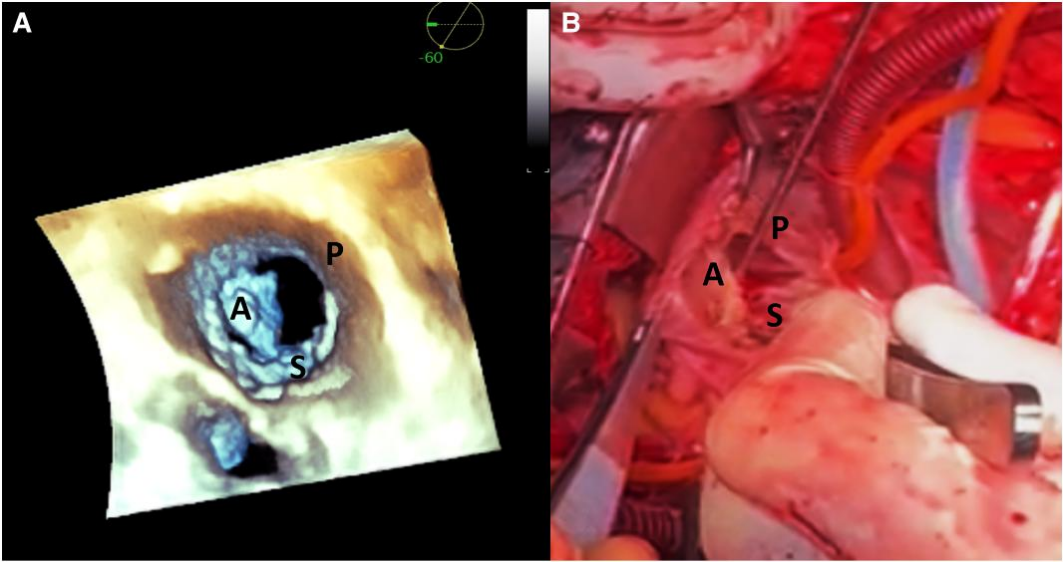
A three-dimensional echocardiogram reveals the following: (*A*) tricuspid valve reconstruction shows the absence of the posterior tricuspid leaflet (surgeon’s view). The marked right heart dilatation allows a particularly clear imaging of the tricuspid valve with transthoracic echocardiography. (*B*) An intra-operative imaging of the tricuspid valve. A, anterior leaflet; P, posterior leaflet; S, septal leaflet.

A transthoracic three-dimensional echocardiogram showed the total absence of the posterior tricuspid leaflet, generating a large regurgitant orifice, a slightly hypoplastic anterior leaflet, and a normal septal leaflet (*Figure 2A*; Supplementary material online, *Videos S3* and *S4*). A transoesophageal echocardiography confirmed the findings (see Supplementary material online, *Videos S5* and *S6*).

Next, a coronary angiogram was performed, which showed normal coronary arteries, while a right heart catheterization revealed a normal pulmonary arterial pressure of 26/6 mmHg and a *Q_p_*/*Q_s_* ratio of 1.

The patient was referred for cardiac surgery after she underwent the standard pre-operative protocol.

Intra-operatively, upon an inspection of the tricuspid valve, it was found that the posterior tricuspid valve leaflet and its subvalvular apparatus were completely absent (*Figure 2B*).

The replacement of the tricuspid valve with a biological prosthesis Medtronic Hancock no. 31 with extracorporeal circulation was carried out with septal leaflet sparring, allowing the protection of the electrical conduction system. The cardiopulmonary bypass time was 145 min and ischaemic time was 81 min. Upon bypass cessation, the patient presented ventricular fibrillation, which was converted to atrial fibrillation after low-energy electrical cardioversion (a single shock of 10 J).

An early post-operative evaluation demonstrated a normally functioning prosthesis with normal transvalvular gradients (maximum/medium = 7/4 mmHg). A mild (Grade I) TR was present. RV function was found moderately impaired (TAPSE = 7–8 mm).

At the 3-month follow-up consultation, the patient reported a significant alleviation of heart failure symptoms (New York Heart Association functional Class II), and the transthoracic echocardiogram showed a normal functioning prosthesis, absence of peri-prosthetic regurgitation, and transvalvular gradients similar to those measured immediately post-surgery. Also, we could observe a partial recovery of RV function, with a calculated TAPSE of 12 mm and a fractional area change of 35%.

## Discussion

We reported the case of an adult patient with a long-standing severe TR of uncertain aetiology up to presentation. In this case, a three-dimensional echocardiography played a crucial role in the evaluation of valve anatomy, allowing the diagnosis of congenital TR and supporting the pre-operative planning of the surgery.

The true prevalence of congenital TR remains undetermined. Historically, the few reported cases were mostly documented during surgery or autopsy.^5^ Currently, the diagnosis of congenital TR is mostly established during foetal echocardiography,^7,8^ and the disorder is associated with high *in utero* and neonatal mortality^7^ due to the development of right heart failure.

In 2011, Gupta *et al*.^5^ proposed a classification of congenital TR based on cuspal (Type 1) or chordal (Type 2) abnormalities, the presence of commissural deficiency (Type 3), annular dilatation (Type 4), or combination (Type 5), or the presence of a double orifice valve (Type 6).

In our case, the patient showed a complex anomaly of the TV with both agenesia of the posterior leaflet and hypoplasia of the anterior leaflet (Type 1). The congenital absence of a tricuspid leaflet is an exceptionally rare congenital disorder, being reported in a fairly small number of cases.^9–14^ While, the agenesis of the anterior leaflet was more commonly described, whereas only in other two cases was the posterior leaflet involved. The pathology needs to be differentiated from the more common aetiology of primary TR—Ebstein’s anomaly and the more extreme form of congenital tricuspid valve dysplasia, such as an unguarded tricuspid valve.^14^

Our patient showed symptoms of advanced right heart failure as well as dyspnoea during minimal exercise. For the latter, the contributing factors could be the interaction of the LV with the severely dilated RV, causing poor compliance of the LV and a lower cardiac output compared with the metabolic demands, or the presence of permanent atrial fibrillation.^15^

While traditionally regarded as a very high-risk surgical procedure, advancements in peri-operative management and improved surgical experience have led to a decline in the mortality of patients undergoing surgery for congenital TR.^16^ Due to the scarcity of data on congenital TR, the optimal surgical approach (tricuspid valve repair or replacement) remains controversial.

The largest evidence regarding the surgical techniques and the post-procedural outcome derives from the population with Ebstein’s anomaly. In such patients, the current guideline favours TV repair by an experienced surgeon over replacement where technically feasible.^17^ Techniques such as cone reconstruction, leaflet augmentation with an autologous pericardium, or/and leaflet reconstruction by plication to leave a bicuspid valve are of choice. Nonetheless, the rate residual TR was shown to be higher after TV repair compared with TV replacement.^18^ Ultimately, more recent data suggest that the choice of surgery (repair vs. replacement) does not influence long-term survival.^16^

With regard to the surgical options in patients with TV agenesia, the existing data are obtained from isolated case reports. Komoda *et al.*^13^ reported a successful repair using a combination of posterior annulorrhaphy and Sebening stitch to reduce the regurgitant orifice. However, in the small number of case reports available, valve replacement was favoured.

In the case of our patient, the complex underlying anatomy did not allow the reconstruction of the valve, and thus, the replacement was favoured. While percutaneous TV replacement has emerged as a therapeutic alternative for severe TR, the procedure seems to be less appropriate for organic TR,^19^ and we did not consider our patient a suitable candidate for the intervention.

## Conclusion

Severe TR was successfully corrected in an adult with the congenital absence of a tricuspid leaflet by prosthetic valve replacement. Post-procedurally, the patient reported a significant relief of symptomatology. This case report illustrates the important role of three-dimensional echocardiography in the diagnosis and management of patients with organic TR.

## Supplementary Material

ytae075_Supplementary_Data

## Data Availability

The data underlying this article will be shared on reasonable request to the corresponding author.

## Lead author biography

Dr Victor C. Raicea graduated from the University of Medicine Târgu Mureș and is currently the head of the Cardiovascular Surgery Department at the University of Medicine and Pharmacy, Craiova. His speciality is valvular surgery and coronary artery disease.

## Notes

**Consent:** The patient has consented to use her medical records and data for scientific purposes, in compliance with COPE guidelines.

**Funding:** No funding.
